# The importance of body weight status on motor competence development: From preschool to middle childhood

**DOI:** 10.1111/sms.13787

**Published:** 2021-04-19

**Authors:** Rodrigo A. Lima, Fernanda C. Soares, Daniel R. Queiroz, Javiera A. Aguilar, Jorge Bezerra, Mauro V. G. Barros

**Affiliations:** ^1^ Institute of Sport Science University of Graz Graz Austria; ^2^ Research Group on Lifestyles and Health University of Pernambuco Recife Brazil

**Keywords:** longitudinal studies, motor development, obesity, youth

## Abstract

We evaluated the association between weight status and motor competence from preschool age (3‐5 years of age) until middle childhood (7‐9 years of age). Longitudinal study with three to five‐year‐old preschool children (n = 1155) enrolled in public and private preschools in Recife, Brazil. Children were followed twice (2010, 2012, and 2014) for four years. Köperkoordinationstest für kinder (KTK) assessed the children's motor competence (KTK Motor Quotient). Weight status (underweight, normal weight, overweight, and obesity) was classified according to the children's sex and age. Preschool children with normal weight exhibited higher motor competence at 5‐7 years of age compared to preschool children with overweight (+3.73 MQ, *P* = .03) and obesity (+5.09 MQ, *P* < .01). Preschool children with normal weight presented higher motor competence at 7‐9 years of age compared to their peers with overweight (+6.00 MQ, *P* = .03) and obesity (+5.88 MQ, *P* = .01). Children with normal weight at 5‐7 years of age presented higher motor competence at 7‐9 years of age compared to their peers with overweight (+3.33 MQ, *P* = .02) and obesity (+4.00 MQ, *P* = .02). Independent of the childhood phase and extension of the period evaluated (2‐ or 4‐year period), children who had excessive weight (overweight or obesity) and changed their weight status to underweight or normal weight presented similar motor competence compared to children who continued underweight or normal weight. Weight status already at preschool age is an important predictor of the children's motor competence until middle childhood. Interventions improving the children's weight status, already at preschool age, might impact their motor competence development positively.

## 1 INTRODUCTION

Engagement in physical activity in the early years of childhood is associated with lower adiposity levels, higher cognitive performance besides improved psychosocial and cardio‐metabolic health.^1^ Furthermore, the ability to move it is an essential aspect to engage in physical activities, especially in early childhood.^2^ Acquiring and refining this movement proficiency involves complex interactions between the environment and the neuromuscular systems, and it has often been referred as motor competence.^3^

A conceptual framework proposed by Stodden et al^4^ suggested a dynamic and synergistic relationship between motor competence, perceived motor competence, physical activity, physical fitness and adiposity during childhood and adolescence. As a result of the publication of this framework, an increasing number of studies have evaluated Stodden's model. Although Stodden et al^4^ acknowledged the possible mutual relationship among perceived motor competence, motor competence, physical activity, physical fitness and adiposity, most of the published evidence have focused on the determinants of adiposity.^5^, ^6^, ^7^

On the other hand, with the rise in the number of young children with overweight and obesity,^8^ studies need to also consider the impact of weight status in young children on the motor competence development during childhood. Children with excessive weight tend to suffer in weight‐bearing activities, which might become a barrier in developing motor competencies.^9^, ^10^, ^11^ Because motor competence during childhood is associated with physical activity and fitness,^5^, ^7^, ^12^, ^13^ evaluating the impact of excessive weight on motor competence development is pivotal. So far, most cross‐sectional^9^, ^10^, ^11^, ^14^, ^15^, ^16^, ^17^, ^18^, ^19^, ^20^ and longitudinal^12^, ^13^, ^21^, ^22^, ^23^ studies reported a negative relationship between weight status or adiposity and motor competence in children. Moreover, longitudinal studies indicate that motor competence and weight status or adiposity might present mutual relationship in children older than 6 years of age.^13^, ^24^ However, most of the manuscripts are cross‐sectional and were conducted with children older than 6 years of age.

It is also relevant to consider the family socioeconomic status while evaluating the association between weight status and motor competence. Veldman et al^25^ reported that lower family income was cross‐sectionally associated with lower motor competence in preschool children. In addition, most aforementioned studies were conducted in high‐income countries (see^6^, ^7^ for review).

Thus, the aims of this study are twofold: to evaluate the association between weight status (underweight, normal weight, overweight and obesity) and motor competence from preschool age (3‐5 years of age) until middle childhood (7‐9 years of age) in Brazilian children; and, to investigate whether weight status trajectory was associated with motor competence during childhood.

## 2 MATERIALS AND METHODS

### 2.1 Procedure

A longitudinal study with preschool children aged three to five years started in 2010. The ELOS‐Pre Project (Longitudinal Study of Health and Well‐being in Preschool Children) was designed to assess longitudinal changes in health conditions, physical activity practices, anthropometric parameters, motor competence performance, and other lifestyle factors among preschool children and in school age. Data were collected by trained graduate and Master's students in physical education. Before all the test rounds, researchers conducted training workshops for all testers. The testers trained their skills on fellow testers and on children in similar age group in a school that was not participating in the ELOS‐Pre Project. After the first data collection that occurred in 2010, children were followed for four years with measurements occurring every second year (2012 and 2014) in Recife, Pernambuco State, Brazil. The protocol was approved by the Human Research Ethics Committee of the University of Pernambuco (protocol no. 0096.0.097.000‐10) and informed written consent was obtained from the children's parents or guardians and the respective school principals.

### 2.2 Participants

**Figure 1:**
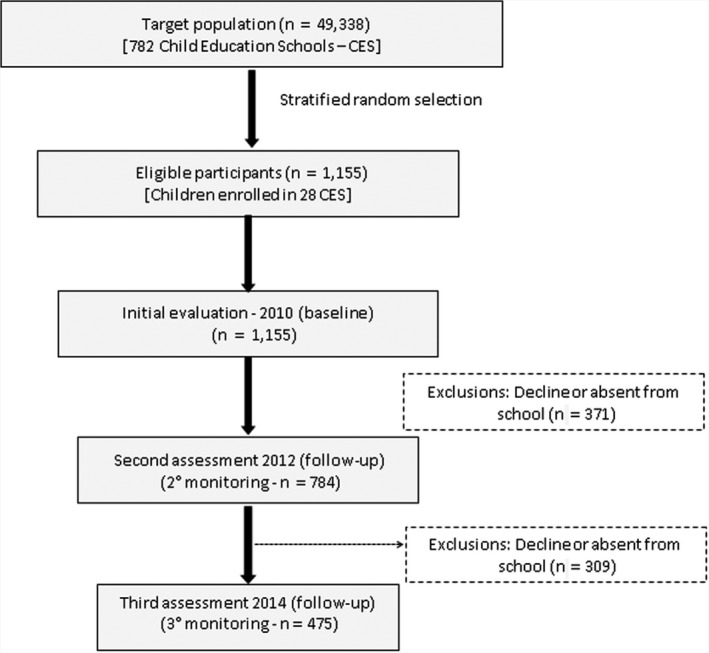
Flow chart of the longitudinal follow‐up of study participants

The target population of the study at baseline was three to five‐year‐old preschool children who were enrolled in both public and private preschools in the six political administrative regions of Recife. Therefore, we selected the schools (cluster unit) to assess the preschool children. All schools in Recife with preschool children were eligible to be included in the study. Stratification criterion was adopted in order to ensure that the sample represented the target population regarding the distribution: type of school (public and private), size of the school (small: <50 children enrolled in preschool education; medium: 50 to 199 children enrolled; large: 200 children or more) and the distribution of the schools according to the six administrative political regions of the city of Recife. In each selected school, all regularly enrolled children were invited to participate in the study.^26^ In total, 1155 children accepted to participate in the baseline (2010). After two years, the project had a follow‐up rate of 76% (n = 784, mean age = 6.0 ± 0.7), and 475 children (mean age = 8.0 ± 0.7) were monitored in 2014 (Figure 1).

### 2.3 Measures

We conducted face‐to‐face interviews with the children's parents to obtain information on their socioeconomic status and health behaviors. Information was collected on sex (male, female), age, and family income.

#### 2.3.1 Motor competence

In 2012 (5‐7 years) and 2014 (7‐9 years), we used the Köperkoordinationstest für kinder (KTK) for directly assessing children's motor competence. The KTK is validated for use in children older than five years of age and consists of 4 independent tests: (a) walking backward on balance beams of decreasing width: 6.0, 4.5, and 3.0 cm; (b) moving sideways on wooden boards for 20 seconds; (c) one‐legged hopping over a foam obstacle with increasing height in consecutive steps of 5 cm; and (d) two‐legged jumping from side to side for 15 seconds. We summed the scores of the four‐abovementioned subtests and used for descriptive purposes (KTK sum of raw scores). For the main analysis, we used the KTK Motor Quotient, which is the sum score of the subtests converted based on normative data of the KTK original study. The KTK Motor Quotient is adjusted for age (all subtests) and gender (hopping for height and jumping sideways over a slat),^27^ and children's motor competence can be classified as impaired motor competence (KTK Motor Quotient 56‐70; ≤2nd percentile), poor motor competence (KTK Motor Quotient = 71‐85; 3rd–16th percentile), normal motor competence (KTK Motor Quotient = 86‐115; 17th–84th percentile), good motor competence (KTK Motor Quotient = 116‐130; 85th–98th percentile), or high motor competence (KTK Motor Quotient 131‐145; ≥99th percentile.^27^

#### 2.3.2 Anthropometric measures

Children were weighed while wearing light clothing with no shoes on a Filizola scale to the nearest 0.1 kg. Their height was measured to the nearest 0.5 cm using a wall‐mounted stadiometer (Welmy, São Paulo, Brazil). Their body mass index (BMI) was calculated by dividing the body weight by the height squared (kg/m^2^). We classified children with underweight, normal weight, overweight, and obesity according to the children's respective sex and age.^28^ Children with overweight or obesity were considered with excessive body weight.

### 2.4 Statistical analyses

All statistical analyses were conducted in STATA 16 for Windows (StataCorp LP). Chi‐square or *t* tests were used to examine differences in the descriptive characteristics between children who were followed in the four‐year monitoring period and children who dropped out.

**Figure 2:**
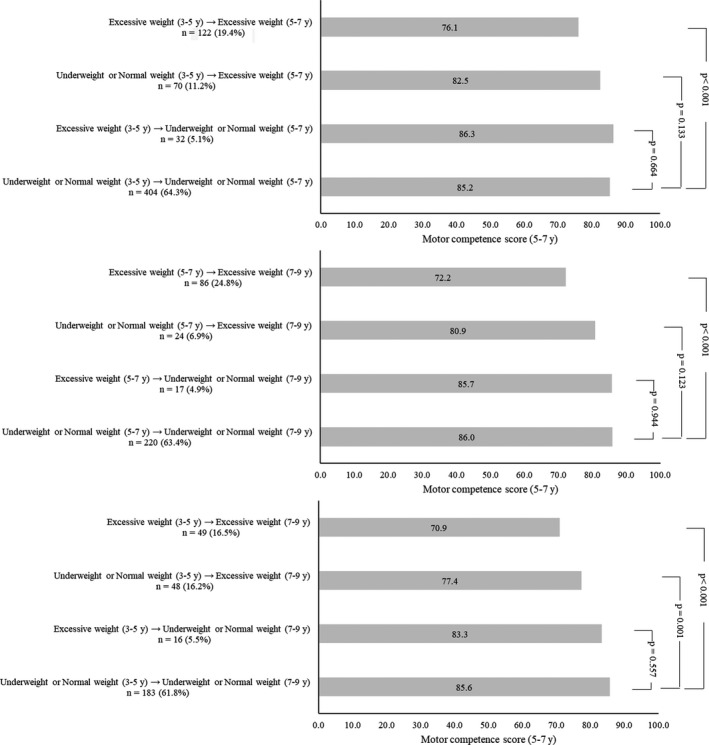
KTK motor Quotient score of the children based on their weight status trajectory during childhood. Legend: Analyses adjusted for family income and the cluster structure of the data (students nested within schools)

Multilevel mixed‐effects linear regressions were used to analyze the associations between weight status and motor competence. Firstly, we evaluated the motor competence depending on the weight status (underweight, normal weight, overweight, and obesity) of the children during childhood (results presented in Table 3). Secondly, we estimated (using margins command in Stata) the motor competence score of the children based on the weight status trajectory (results presented in Figure 2). As an example, we calculated the motor competence score of the children at 5‐7 years of age who presented excessive weight at preschool age (3‐5 years) and continued with excessive weight in the first follow‐up at 5‐7 years of age. We provide detailed description of the adjustments for each model in the results section, in the respective Tables and Figures legends. In all the multilevel regression models, the variance related to the clusters (school) and the intraclass correlation coefficient (ICC) for each model was calculated to interpret the variation among schools and individuals. In all the regression models, the majority of the variation (ICC) was at the individual level (the ICCs from the schools were always below 3%).

## 3 RESULTS

Table 1 presents descriptive data of the participants followed in each of the measurements and of the participants not followed in 2012 or 2014. We did not observe selection bias in relation to the family income and the children's sex, weight status, and age (all *P* > .05) by comparing children who dropped out in 2012 or in 2014 with children assessed in 2010 who continued to be monitored in 2012 and 2014. In 2010, on average, children were 4.3 years of age, 51.4% were boys and 26.2% had excessive body weight.

**Table 1:** Sociodemographic characteristics and body measurements of the participants at the baseline (2010), follow‐ups (2012, 2014), and the dropouts

	Variables	2010 (3‐5 y)		2012 (5‐7 y)		2014 (7‐9 y)		Dropouts (2012 or 2014)		*P*
		n	%	n	%	n	%	n	%	
Sex	Male	594	51.4	405	51.7	230	52.3	364	61.3	.652^a^
	Female	561	48.6	379	48.3	210	47.7	351	62.6	
Family income	<1 MW	425	42.4	287	37.1	154	39.6	283	64.2	.126^b^
	1‐2 MW	307	29.6	247	32.0	111	28.5	216	71.4	
	2‐4 MW	152	14.6	129	16.7	68	17.5	104	68.4	
	>4 MW	139	13.4	110	14.2	56	14.4	85	61.2	
BMI	Underweight	166	14.4	74	9.9	43	10.8	111	66.9	.223^b^
	Normal weight	645	55.8	445	59.2	229	57.5	414	64.2	
	Overweight	153	13.3	124	16.5	74	18.6	111	72.6	
	Obesity	191	16.5	108	14.4	52	13.1	121	63.4	

		n	mean (SD)	n	mean (SD)	n	mean (SD)	n	mean (SD)	
Age (y)		1555	4.3 (0.8)	783	6.3 (0.8)	440	8.4 (0.8)	715	4.3 (0.8)	.125^c^

Abbreviation: MW, minimum wages.

^a^ *P* value from the Fisher chi‐square test comparing dropouts and children monitored in 2010.

^b^ *P* value from the Exact Fisher chi‐square test comparing dropouts and children monitored in 2010.

^c^ *P* value from the dependent t test comparing dropouts and children monitored in 2010.

In 2012 (5‐7 years of age), 18.2% children presented impaired motor competence, 39.3% poor motor competence, 40.8% normal motor competence, 1.6% good motor competence, and 0.1% high motor competence. In 2014 (7‐9 years of age), 22.0% children presented impaired motor competence, 35.7% poor motor competence, 40.2% normal motor competence, 2.1% good motor competence, and none high motor competence. Table 2 describes the children's motor competence scores for each of the KTK tasks, for the KTK sum of raw scores and for the KTK Motor Quotient.

**Table 2:** Participants' motor competence scores during childhood

Motor competence (scores)	2012 (5‐7 y)		2014 (7‐9 y)	
	n	Mean (SD)	n	Mean (SD)
KTK sum of raw scores	746	101.9 (34.8)	440	146.5 (41.2)
KTK Motor Quotient	746	83.4 (14.5)	440	82.2 (16.4)
KTK tasks (standardized scores)				
Walking backward	747	89.4 (16.0)	451	94.3 (15.7)
Moving sideways	751	94.6 (17.5)	460	88.0 (17.8)
One‐legged hopping	751	72.9 (13.3)	461	76.1 (17.6)
Two‐legged jumping	750	92.1 (13.5)	461	87.7 (15.2)

We observed that weight status and motor competence were cross‐sectionally and longitudinally associated throughout childhood (Table 3). Overall, children who were overweight or obese presented lower motor competence score during childhood compared to children who were normal weight. In summary, preschool children with normal weight exhibited higher motor competence at 5‐7 years of age compared to preschool children with overweight (+3.73 MQ points, *P* = .03) and obesity (+5.09 MQ points, *P* < .01). Preschool children with normal weight presented higher motor competence at 7‐9 years of age compared to their peers with overweight (+6.00 MQ points, *P* = .03) and obesity (+5.88 MQ points, *P* = .01).

**Table 3:** Longitudinal associations between weight status and motor competence scores during childhood

Exposures		KTK motor Quotient score at 5‐7 y (2012)			KTK motor Quotient score at 7‐9 y (2014)		
		n (%)	Mean difference in relation to normal weight (95% CI)	*P*	n (%)	Mean difference in relation to normal weight (95% CI)	*P*
BMI at 3‐5 y (2010)	Underweight	107 (16.2)	2.96 (−0.13 to 6.05)	.06^a^	52 (14.8)	5.95 (1.16 to 10.74)	.02^b^
	Overweight	83 (12.5)	−3.73 (−7.16 to −0.31)	.03^a^	39 (11.1)	−6.00 (−11.39 to −0.62)	.03^b^
	Obesity	98 (14.8)	−5.09 (−8.28 to −1.89)	<.01^a^	55 (15.7)	‐5.88 (−10.57 to −1.20)	.01^b^
BMI at 5‐7 y (2012)	Underweight	74 (10.1)	1.51 (−1.90 to 4.92)	.39^c^	35 (9.2)	2.83 (−0.78 to 6.44)	.12^d^
	Overweight	121 (16.4)	−4.26 (−7.06 to −1.45)	<.01^c^	73 (19.2)	−3.33 (−6.07 to −0.60)	.02^d^
	Obesity	106 (14.4)	−11.12 (−14.07 to −8.17)	<.01^c^	49 (12.9)	−4.00 (−7.30 to −0.70)	.02^d^
BMI at 7‐9 y (2012)	Underweight				40 (11.3)	2.83 (−2.26 to 7.91)	.28^e^
	Overweight				68 (19.3)	−6.53 (−10.66 to −2.39)	<.01^e^
	Obesity				44 (12.5)	−19.81 (−24.72 to −14.89)	<.01^e^

Note

that all the models were adjusted for the cluster structure of the data (students nested within schools).

^a^ Adjusted for: family income (3‐5 and 5‐7 y).

^b^ Adjusted for: family income (3‐5 and 7‐9 y).

^c^ Adjusted for: family income (5‐7 y).

^d^ Adjusted for: KTK motor Quotient score (5‐7 y), family income (5‐7 and 7‐9 y).

^e^ Adjusted for: family income (7‐9 y).

Children with normal weight at 5‐7 years of age presented higher motor competence at 5‐7 years of age compared to their peers with overweight (+4.26 MQ points, *P* < .01) and obesity (+11.12 MQ points, *P* < .01). Children with normal weight at 5‐7 years of age presented higher motor competence at 7‐9 years of age compared to their peers with overweight (+3.33 MQ points, *P* = .02) and obesity (+4.00 MQ points, *P* = .02). Children with normal weight at 7‐9 years of age presented higher motor competence at 7‐9 years of age compared to their peers with overweight (+6.53 MQ points, *P* < .01) and obesity (+19.81 MQ points, *P* < .01) (Table 3).

Figure 2 presents the motor competence score of the children at 5‐7 and at 7‐9 years of age based on the weight status trajectory during childhood. In summary, independent of the childhood phase and extension of the period evaluated (two‐ or four‐year period), children who had excessive weight (overweight or obesity) and changed their weight status to underweight or normal weight presented similar motor competence scores compared to children who continued underweight or normal weight. As an example, preschool children with excessive weight who changed their weight status to underweight or normal weight at 5‐7 years of age presented similar motor competence score at 5‐7 years of age compared to children who continued in the underweight or normal weight groups in the same period (*P* = .664).

## 4 DISCUSSION

We observed that motor competence scores throughout childhood were dependent on the children's weight status. Interestingly, even weight status at preschool age‐predicted motor competence scores four years later. To the best of our knowledge, this is the first longitudinal study evaluating the relationship between weight status at preschool age and motor competence scores in early and middle childhood. In general, previous longitudinal investigations reported a deleterious impact of weight status on motor competence during childhood.^12^, ^13^, ^22^, ^29^

It is believed that children with excessive weight experience more difficulties in weight‐bearing activities because of the greater weight that must be beared against gravity during these tasks.^9^, ^10^, ^11^ In addition to the biomechanical limitations related to excessive weight, children with higher adiposity or excessive weight are less engaged in physical activities^30^ and also present lower physical fitness.^5^, ^13^ Heavier children are less likely to participate in physical activities, which in turn diminishes the number of opportunities for developing their motor competence and fitness levels.^13^ This causal cycle is likely to increase the children's weight status and feedback this vicious cycle.^4^

Our results provide new insights in the Stodden's model by showing the importance of weight status already at preschool age on the motor competence development during childhood. Although Stodden et al^4^ acknowledged the importance of weight status on the motor competence, perceived motor competence, physical and fitness developments, Stodden's model suggested that participation in physical activities and motor competence would be the most important components in early childhood. A growing body of evidence is highlighting the importance of weight status already at very young ages.^31^, ^32^, ^33^ Nevertheless, future investigations need to provide additional information for better understanding. This is a crucial aspect that needs further investigation to support the design of interventions targeting health behaviors in children.

Moreover, independent of the period evaluated, children who changed their weight status from excessive weight to underweight or normal weight presented similar motor competence scores compared to children who continued underweight or normal weight within the two‐ or four‐year period, which is a novel finding. Although children with excessive weight tend to have lower motor competence,^12^, ^22^, ^29^ interventions improving their weight status might also enhance their motor competence.

Although the current investigation presents novel findings regarding the importance of weight status on the motor competence development, few limitations need to be considered in the interpretation of the findings. First, the KTK battery is only valid for use in children older than 5 years of age, thus we did not assess children's motor competence at preschool age. Nevertheless, the interpretation regarding the importance of weight status on the children's motor competence remained similar in the models in which we were able to adjust for the KTK scores. Second, this was a longitudinal study; therefore, we were not able to infer causality in the weight status‐motor competence relationship.

Our findings support the importance of weight status already at preschool age on the motor competence development of children until middle childhood. Moreover, our results support that changes in the weight status are associated with changes in motor competence scores. Therefore, we recommend that future interventions test the impact of changes in the children's weight status on their motor competence development.

## 5 PERSPECTIVE

Weight status, as early as preschool age, predicted motor competence scores in early (5‐7 years) and middle (7‐9 years) childhood. In addition, we observed that changes in weight status during childhood were related to the children's motor competence scores. Although the current findings highlight the importance of weight status already at young ages on the motor competence development, it is feasible that both motor competence and weight status are mutually related and equally important during childhood development.^13^ Therefore, interventions should target both weight status and motor competence, since their probable synergic relationship stimulates one another.

## CONFLICT OF INTEREST

None to declare.

## AUTHOR CONTRIBUTIONS

Lima RA contributed to conception, design, data acquisition, analysis, and interpretation; he also drafted and critically revised the manuscript. Queiroz DR, Aguilar JA, and Bezerra J contributed to data acquisition, analysis, and interpretation; they also drafted and critically revised the manuscript. Soares FC and Barros MVG contributed to design, data analysis, and interpretation; they also drafted and critically revised the manuscript. All authors gave their final approval of the text and agree to be accountable for all aspects of the work.

## ETHICAL APPROVAL

Where research involves human and/or animal experimentation. The following statements should be included (as applicable): “The authors assert that all procedures contributing to this work comply with the ethical standards of the relevant national and institutional committees on human experimentation and with the Helsinki Declaration of 1975 as revised in 2008.” and “The authors assert that all procedures contributing to this work comply with the ethical standards of the relevant national and institutional guides on the care and use of laboratory animals.”
